# The Excised Appendix Tip—To Send or not to Send, That is the Question

**DOI:** 10.1055/s-0038-1672166

**Published:** 2018-10-18

**Authors:** Lucinda Tullie, Bhumita Vadgama, Ravindar Anbarasan, Michael P. Stanton, Henrik Steinbrecher

**Affiliations:** 1 Department of Paediatric Surgery and Urology, Southampton Children's Hospital, Southampton, United Kingdom of Great Britain and Northern Ireland; 2 Department of Histopathology, University Hospital Southampton NHS Foundation Trust, Southampton, United Kingdom of Great Britain and Northern Ireland

**Keywords:** appendix, carcinoid, pediatric, antegrade continence enema

## Abstract

A 9-year-old boy, with previous anorectal malformation and neuropathic bladder and bowel, underwent ileocystoplasty, Monti–Mitrofanoff and appendix antegrade colonic enema procedure. The tip of the macroscopically normal appendix was sent for routine histopathology. Microscopy demonstrated a 5-mm well-differentiated neuroendocrine tumor extending into muscularis propria. K _i_ -67 index was <2%. Due to margin involvement, the appendix conduit and surrounding skin were re-excised and a tube cecostomy was created through a separate incision. Microscopy revealed no residual neuroendocrine tumor, and no further treatment was required.

## Introduction

Since it was first described in the early 1980s, first by Mitrofanoff and then by Malone (antegrade continence enema [ACE]) the appendix has been used as a catheterizable conduit, for both bladder and bowel. 1 2 Although use of ileum, ureter, and even fallopian tube has also been described, the appendix remains the most commonly used conduit.

We describe a case of incidental carcinoid tumor in the tip of an appendix used for an ACE procedure.

## Case Report

A 9-year-old boy, with previous anorectal malformation, corrected with a posterior sagittal anorectoplasty (PSARP), developed neuropathic bladder and bowel. He underwent an ileocystoplasty and Monti–Mitrofanoff and appendix ACE procedure. The tip of the macroscopically normal appendix was sent for routine histopathology, which is a standard practice for the operating surgeon.

**Fig. 1:**
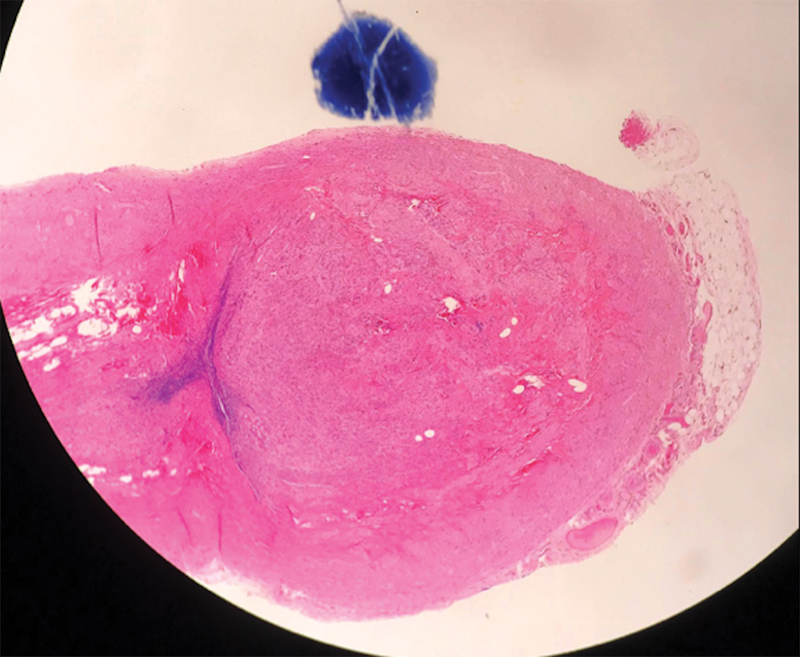
H&E stain demonstrating a mass at the tip of the appendix. H&E, hematoxylin and eosin.

**Fig. 2:**
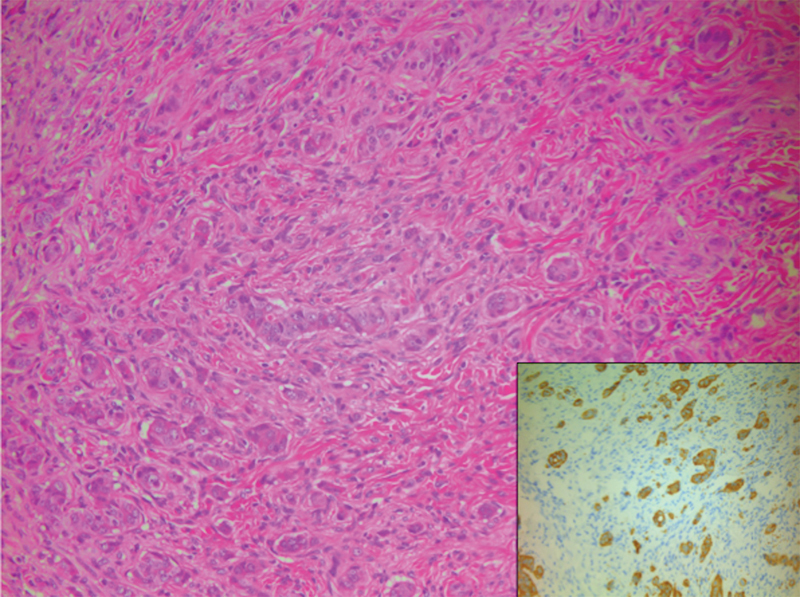
H&E stain demonstrating infiltrating neuroendocrine cells and nests confirmed by positive synaptophysin staining (inset). H&E, hematoxylin and eosin.

Microscopy demonstrated a 5-mm well-differentiated neuroendocrine tumor in the tip of the appendix ( Fig. 1 ), which extended into muscularis propria. K _i_ -67 tumor proliferation index was <2%. On hematoxylin and eosin (H&E) staining, nests of neuroendocrine cells could be seen infiltrating into the appendix wall and involved the proximal resection margin, and findings were confirmed on synaptophysin staining ( Fig. 2 ).

Due to proximal margin involvement and following neuroendocrine tumor multidisciplinary team (MDT) recommendation, the appendix conduit and surrounding skin was re-excised and a tube cecostomy was created through a separate incision. Microscopy of the remainder of the appendix revealed no residual neuroendocrine tumor, and no further treatment was required. The patient remained well at the last follow-up.

## Discussion

Neuroendocrine or carcinoid tumors, first described in 1867 by a Swiss pathologist Theodore Langhans, are most frequently found in the gastrointestinal tract with two-thirds located in the appendix. 3 In children, carcinoid most commonly presents with symptoms suggestive of appendicitis or is detected when the appendix, or portion of appendix, is removed incidentally during surgery, as was the case for our patient. 3

The incidence of appendix carcinoid in children is variable, reported in between 0.08 and 0.7% of appendicectomies. 3 4 Even in symptomatic cases, carcinoid tumors are rarely identified at the time of surgery. A recent systematic review of pediatric carcinoid tumors found that in 91% cases, carcinoid was only identified following histology, as we report in our case. 5 The majority of studies in this review report cases of appendectomies undertaken for symptomatology, acute appendicitis or chronic abdominal pain with a carcinoid incidence of 0.3% of appendectomies. 5 Incidental appendectomies, performed in an adult population of patients with normal appendices, demonstrated a much higher carcinoid incidence of 1.6%. 6

To our knowledge, this is only the second reported pediatric case in the literature of incidental carcinoid in an appendix conduit. The first case, reported in the late 1990s, was in a 7-year old girl who had an appendix Mitrofanoff. 7 Similar to our case, the appendix appeared macroscopically normal at operation and, with the exception of excision of the residual appendix and revision of the Mitrofanoff, the patient also required no further treatment.

While the literature reports a variety of techniques for Mitrofanoff and ACE formation, none mentions whether the appendix conduit tip should be sent for histopathology. However, the majority of United Kingdom surgeons we surveyed do not request routine histology on the appendix tip when using it to form a catheterizable conduit.

## Conclusion

Appendix carcinoid is most commonly diagnosed at histology and may be detected without prior symptomatology. Appreciating this, and with the recognized, albeit small, risk of carcinoid in the appendix conduit, we therefore recommend routine histological examination of the appendix tip.

## Notes

New Insights and the Importance for the Pediatric Surgeon The majority of surgeons using the appendix as a catheterizable conduit do not send the appendix tip for histology. This is the first reported case of carcinoid in an appendix antegrade colonic enema and the second reported case of appendix carcinoid in an appendix used as a catheterizable conduit.
